# Evidence for magnesium–phosphorus synergism and co-limitation of grain yield in wheat agriculture

**DOI:** 10.1038/s41598-021-88588-8

**Published:** 2021-04-27

**Authors:** Martin Weih, Hui Liu, Tino Colombi, Thomas Keller, Ortrud Jäck, Pernilla Vallenback, Anna Westerbergh

**Affiliations:** 1 Department of Crop Production Ecology, Swedish University of Agricultural Sciences, PO Box 7043, 750 07 Uppsala, Sweden; 2 Department of Soil and Environment, Swedish University of Agricultural Sciences, 750 07 Uppsala, Sweden; 3 Department of Agroecology and Environment, Agroscope, 8046 Zürich, Switzerland; 4 Lantmännen Lantbruk, 268 31 Svalöv, Sweden; 5 Department of Plant Biology, Swedish University of Agricultural Sciences, 750 07 Uppsala, Sweden

**Keywords:** Agroecology, Ecophysiology, Plant ecology

## Abstract

Modern crop production is characterized by high nitrogen (N) application rates, which can influence the co-limitation of harvested yield by other nutrients. Using a multidimensional niche volume concept and scaling exponents frequently applied in plant ecological research, we report that increased N and phosphorus (P) uptake in a growing wheat crop along with enhanced grain biomass is associated with more than proportional increase of other nutrients. Furthermore, N conversion efficiency and grain yield are strongly affected by the magnesium (Mg) to P ratio in the growing crop. We analyzed a field trial in Central Sweden including nine wheat varieties grown during two years with contrasting weather, and found evidence for Mg co-limitation at lower grain yields and P co-limitation at higher yields. We argue that critical concentrations of single nutrients, which are often applied in agronomy, should be replaced by nutrient ratios. In addition, links between plant P and Mg contents and root traits were found; high root number enhanced the P:N ratio, whilst steep root angle, indicating deep roots, increased the Mg:N ratio. The results have significant implications on the management and breeding targets of agriculturally grown wheat, which is one of the most important food crops worldwide.

## Introduction

Proper plant growth requires many nutrient elements, of which nitrogen (N) and phosphorus (P) are considered quantitatively most important^1,2^. Most research on plant nutrient uptake and use efficiency has therefore focused on N and P, although also other nutrients can play a significant role in (co-)limiting plant growth and crop production^3–5^. As N is often limiting crop growth, modern crop production systems are typically characterized by high N application rates, and commercial fertilizers in agriculture frequently contain N, P and K (potassium) but little or no other nutrients. Theoretical considerations suggest that all essential elements for growth and yield formation should scale in proportion to each other (homeostasis)^6^. Deviations from proportionality could indicate that those nutrients that are accumulated less than proportionally become growth or yield limiting, which might become critical particularly under well-fertilized conditions supporting increased growth and yield. It is therefore possible that a high N and P application rate results in co-limitation of crop growth and yield by other nutrients. In general, the scaling relationships between many morphological plant traits appear to be conserved, and this applies also to the tissue concentrations of elements that are essential for metabolic reactions involved in photosynthesis, biomass growth and yield formation^7^. Assuming that conserved element scaling relationships result in restricted nutrient uptake in certain growth conditions, restricted plant-internal nutrient availabilities could become (co-)limiting plant carbon acquisition and growth. The evaluation of scaling relationships for nutrient elements is often done by quantifying shifts in element ratios, which is feasible as long as the analysis is restricted to few nutrient elements. When many nutrient elements are involved, metrics based on the multidimensional niche volume concept^8^ can be used. Focusing on many nutrient elements, a niche volume concept was applied to various plants including wheat to analyze the relationships between the vegetative tissue concentrations of N and P (expressed as their volume, VNP) and the concentrations of other macro nutrients (VOth)^9^. Based on the scaling relationship VOth = β(VNP)^α^, a faster increase of VOth in relation to VNP is reflected by scaling exponents (α) > 1, whilst a slower increase of VOth in proportion to VNP is reflected by scaling exponents < 1. The regressions describing the scaling relationships in this type of studies are usually calculated as reduced major axes (RMA)^10^. Scaling exponents have also been calculated for the relationship between N and P (e.g., [P] = β[N]^α^) and linked to the growth rates for various plants^7,10^, but not to harvestable yields of major crops such as wheat. More importantly, scaling exponents considering nutrients other than N and P have not been studied in relation to crop yields. Scaling exponents between N and P < 1, i.e. P accumulating slower than N, have been reported as a general rule for most plants^7^. At the same time, increased efficiency of P acquisition especially during early crop development is today considered an important breeding target because P has been found to limit plant growth and crop production in many cases^11–14^. There are also indications of magnesium (Mg) co-limitation in crop production, because low Mg concentrations in leaves can result in reduced photosynthetic N productivity and ultimately crop yield, and Mg fertilization has been shown to increase crop yields^15,16^. However, studies attempting to establish critical Mg concentrations for optimal plant growth often remain inconclusive and do not consider the interaction with other growth-limiting nutrients^16^.

Especially for spring wheat grown under the short growing seasons at higher latitudes, early growth of roots and leaves from seed to tillering (early vigor), and the root characteristics during this period, are considered important for nutrient uptake and ultimately grain yield formation^17^. In addition, the nutrient composition of the growing crop can greatly vary during the different growth stages and also depending on soil and weather conditions^18,19^. However, clear links between root characteristics, nutrient composition beyond N and P, and growth and yield are lacking for wheat and most other plants. Some links between specific root characteristics and nutrient acquisition have been established for N and P^20–24^, but rarely for other nutrients. For example, root angles tend to become steeper when roots are growing deeper in low N conditions^25^, suggesting that root characteristics could be linked to the vertical distribution of specific nutrients in soil. It has also been shown that the vertical distribution in soil varies between nutrient elements; e.g., P has often a shallower vertical distribution than Mg^26^, suggesting that increased Mg uptake possibly could be associated with deeper roots (or steeper root angles) whilst increased topsoil rooting may enhance P acquisition^27^. The combined analysis of scaling exponents, root characteristics and grain yields offers a unique possibility to establish links between nutrient availability in soil, plant root characteristics, element proportions beyond N and P, plant growth and grain yield.

**Figure 1:**
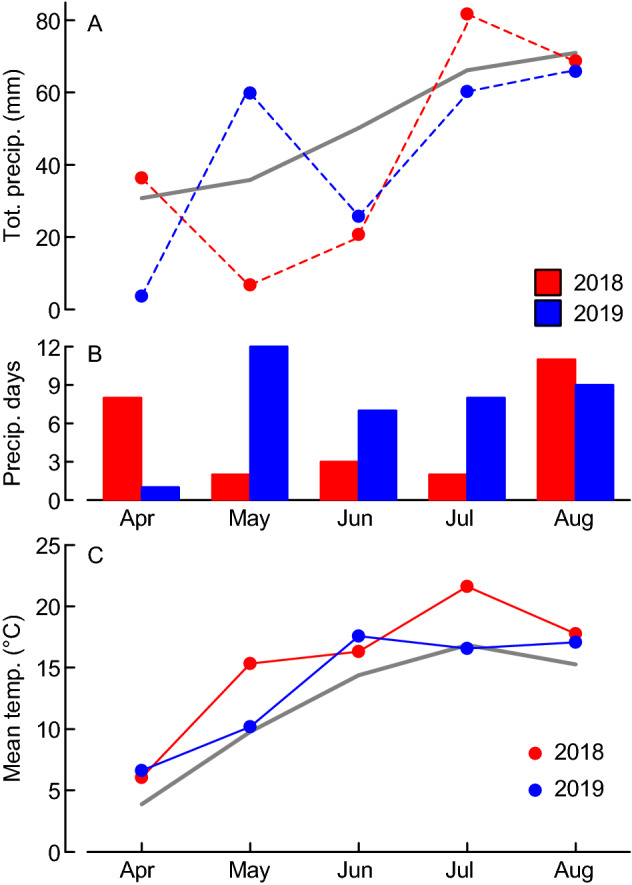
Weather conditions during the experimental periods of the years 2018 and 2019. (**A**) Total monthly precipitation, (**B**) number of days with precipitation ≥ 1 mm, and (**C**) mean air temperature. Long-term (1896–2019) means (solid gray lines) are plotted for comparison. All data were collected at the Ultuna climate station near Uppsala, situated 3 km south-west from the experimental site.

The major aim of this study was to relate the proportional changes in the vegetative tissue concentrations of some macro (Ca, K, Mg, S) and micro nutrients (Cu, Fe, Mn, Zn) based on the concentrations of N and P (reflected by scaling exponents) to grain yields and N conversion efficiency. Furthermore, we aimed at identifying critical nutrient elements for co-limitation of grain yield; and at linking their uptake to juvenile root and shoot growth (early vigor) on the one hand, and grain yield on the other hand. The aims were addressed by exploring a data set from field-grown spring wheat in which variation in nutrient concentrations and grains biomass was created using nine contrasting varieties grown at two soil compaction treatments during two years with contrasting weather conditions (Fig. 1; Table 1). Based on the biomass and nutrient data assessed, we calculated RMA scaling exponents according to a standard methodology^9^; and grain-specific N efficiency (E_N,g_) in terms of the grains biomass per growing-season mean N pool^28^ as an indicator of N conversion efficiency. Published element concentration and grain yield data from additional Swedish winter wheat trials^19^ were used to validate the results for a different crop type (winter wheat), soils and fertilization conditions.

**Table 1:** Soil properties and soil nutrient status (ammonium acetate–lactate (AL) extractable P, K and Mg) at the experimental site in 2018 and 2019.

Property	2018	2019
Clay (g kg^−1^)	145	145
Total N (g kg^−1^)	2.4	2.3
Total soil organic C (g kg^−1^)	25.9	24.7
pH (H_2_O)	5.8	5.7
P–AL (g kg^−1^)	0.034	0.038
K–AL (g kg^−1^)	0.109	0.110
Mg–AL (g kg^−1^)	0.194	0.201

The soil was sampled in 0–0.3 m depth after the application of fertilization, and the 5 sampling points were different in 2018 and 2019. Fertilization with 140 kg ha^−1^ N, 24 kg ha^−1^ P and 46 kg ha^−1^ K was applied directly after sowing in both years. The soil organic carbon content was measured using dry combustion and infrared gas analysis (LECO).

## Results

### P accumulated slower than N but the concentrations of other nutrients increased faster than the concentrations of N and P

**Figure 2:**
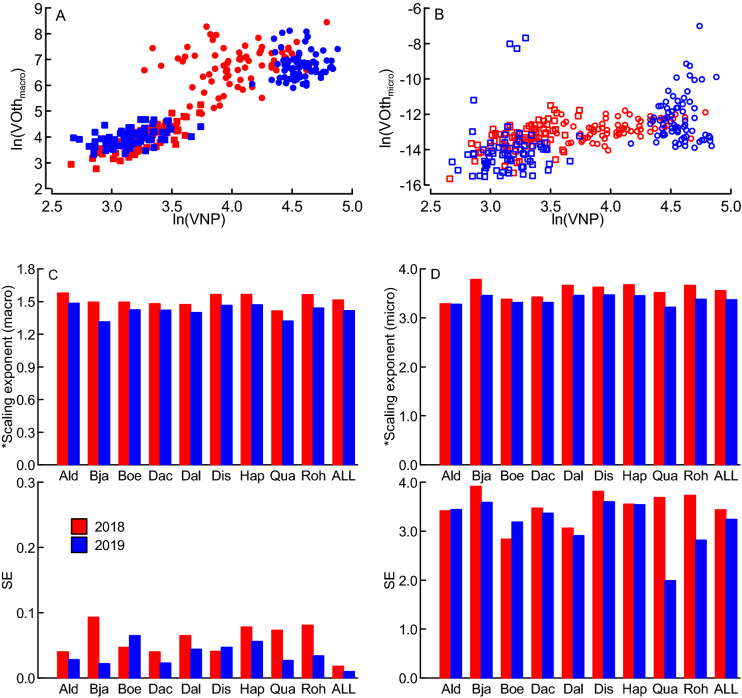
Scatter plots of the volumes (natural logarithm) of plant N and P concentrations (VNP) versus the volumes of other elements (VOth) at tillering (circles) and flowering (squares) (**A** macroelements, closed symbols; **B** microelements, open symbols); and the corresponding scaling exponents (means and standard errors, SE, from 4 replicates; **C**, **D**) for nine wheat varieties field-grown in Sweden during two years (2018 red, 2019 blue). Data from two soil compaction treatments were included, but not separately presented as no significant differences were found between them. (**A, C**) VOth = macroelements (Ca, K, Mg, S) and (**B, D**) VOth = microelements (Cu, Fe, Mn, Zn). For the macroelements, the scaling exponents differed significantly between varieties (ANOVA, *N* = 16, *F* = 13.87, *P* < 0.001) and years (*N* = 144, *F* = 23.12, *P* < 0.001) but not compaction treatments (*N* = 144, *F* = 3.14, *P* = 0.077); for the microelements, scaling exponents did not significantly differ between varieties, years or treatments (ANOVA, *P* > 0.05). Varieties **Ald** ‘Alderon’, **Bja** ‘Bjarne’, **Boe** ‘Boett’, **Dac** ‘Dacke’, **Dal** ‘Dala landrace’, **Dis** ‘Diskett’, **Hap** ‘Happy’, **Qua** ‘Quarna’, **Roh** ‘Rohan’; **ALL** indicates the means and SE across all varieties. * indicates scaling exponents based on data from tillering (BBCH29) and flowering (BBCH65)^35^. *N* = 288 in (A) and (B), where the data represent individual replicates.

Across all varieties, years and developmental stages (tillering and flowering), P accumulated slower than N (RMA scaling exponent 0.193, standard error = 0.004). As expected, the concentration volumes of other nutrients (VOth) increased with the volumes of N and P (VNP) in both years, and these concentration volumes were generally higher at tillering than flowering stage (Fig. 2A,B). RMA scaling exponents for both macro (VOth_macro_; Ca, K, Mg, S) and microelements (VOth_micro_; Cu, Fe, Mn, Zn) were clearly > 1; i.e., the two groups of nutrients other than N and P accumulated faster than N and P.

### Great variation in scaling exponents and other traits between varieties and years

The scaling exponents for VOth_macro_ vs. VNP varied significantly between years and varieties, but were similar for the two soil compaction treatments (Fig. 2C and statistical information therein). Also agronomic grain yield (G_Y_) varied significantly between years and varieties, but was not significantly affected by the soil compaction treatment (Supplement S1). All scaling exponents (macronutrients) were greater in the drier and warmer year (2018) compared to the wetter and cooler year (2019), implying faster accumulation of nutrients other than N and P in the drier and warmer weather conditions (Fig. 2C). In addition, the higher-yielding varieties (e.g., ‘Alderon’ and ‘Happy’) had greater scaling exponents (macronutrients) than the lower-yielding varieties (Fig. 2C and Supplement S2A). The scaling exponents for VOth_micro_ vs. VNP were high and showed large variability (Fig. 2D); and were not significantly affected by any of the factors investigated. Grains biomass, yield-specific N efficiency (E_N,g_), root traits and nutrient concentrations varied significantly between years and varieties, but were indifferent between the two soil compaction treatments (ANOVA, Supplement S1). Due to the paucity of significant effects of the soil compaction treatment on the response variables investigated, we used the data from the two compaction treatments only to verify the lack of any treatment effect on scaling exponents, and performed all further data analysis on the data from the non-compacted (control) treatment only. According to Principal Components Analysis (PCA, Supplement S3), 75% (dim. 1) of the variation in the data was explained by year effects, whilst 14% (dim. 2) was explained by variation between varieties.

### Early-season Mg:N and P:N ratios were linked to root traits and early vigor

**Figure 3:**
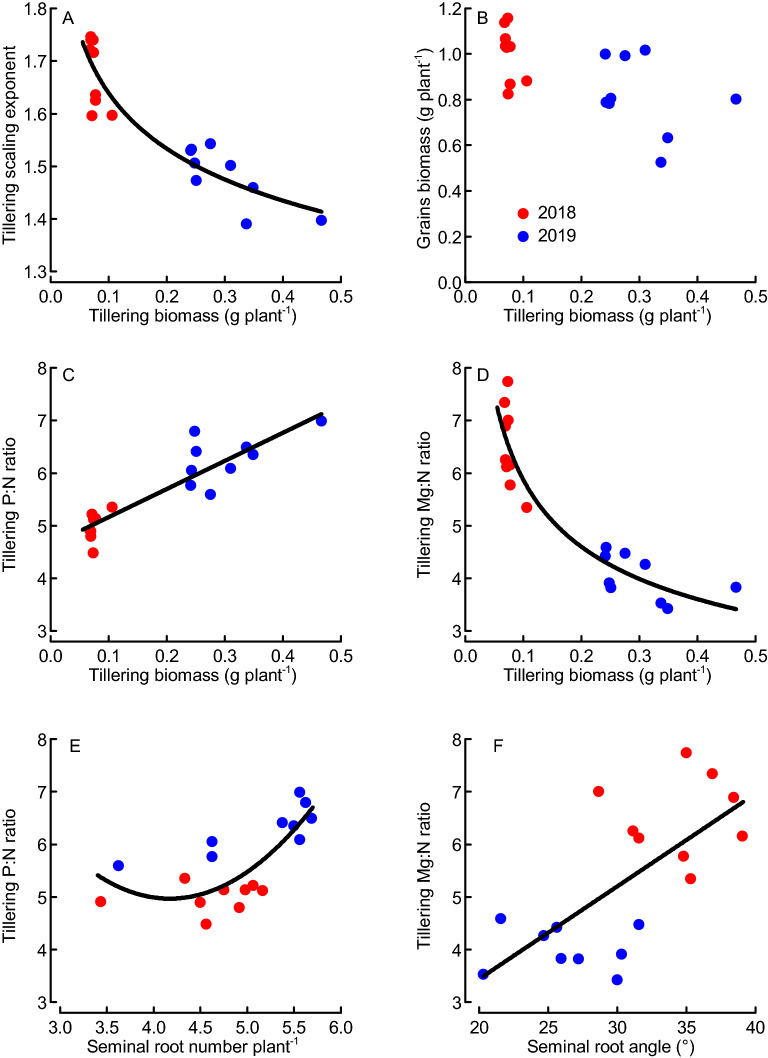
Scaling exponent (macroelements), nutrient ratios and harvested grains biomass as functions of early vigor biomass and root traits (tillering, BBCH29)^35^ across nine wheat varieties field-grown in Sweden during two years (2018, 2019). Regressions: y = 1.314x^−0.096^, *N* = 18, *R*^2^ = 0.86, *P* < 0.001 (**A**); y = 5.340x + 4.630, *N* = 18, *R*^2^ = 0.82, *P* < 0.001 (**C**); y = 2.611x^−0.352^, *N* = 18, *R*^2^ = 0.89, *P* < 0.001 (**D**); y = 0.744x^2^ − 6.210x + 17.927, *N* = 18, *R*^2^ = 0.61, *P* = 0.001 (**E**); y = 0.176x − 0.078, *N* = 18, *R*^2^ = 0.47, *P* = 0.002 (**F**). Dots represent means from 4 replicates.

Across both years, RMA scaling exponents (VOth_macro_ vs. VNP) decreased with increasing plant biomass at tillering, but not within 2018 where all varieties had similar biomass but different scaling exponents (Fig. 3A). Plant biomass at tillering (early vigor) was a poor predictor for grains biomass (Fig. 3B; linear regressions within years, *P* ≤ 0.155 n.s., *N* = 9). Among all macro nutrients (Ca, K, Mg, S), the RMA scaling exponent was most strongly associated with the Mg:N ratio (correlation: Pearson *R* = 0.95, *N* = 18, *P* < 0.001) and P:N ratio (Pearson *R* = − 0.93, *N* = 18, *P* < 0.001). The P:N ratio increased and the Mg:N ratio decreased with increasing tillering biomass (Fig. 3C,D). When evaluated across years, the P:N ratio increased with the seminal root number above a value of ca. 4 roots plant^−1^, whilst the Mg:N ratio increased with steeper seminal root angle (Fig. 3E,F). Seminal root number and angle were negatively correlated (Pearson *R* = − 0.53, *N* = 18, *P* = 0.024), indicating that root growth in the topsoil increased with higher seminal root number.

### Root traits affected scaling exponent and Mg:P ratio, which in turn influenced the grain number

**Figure 4:**
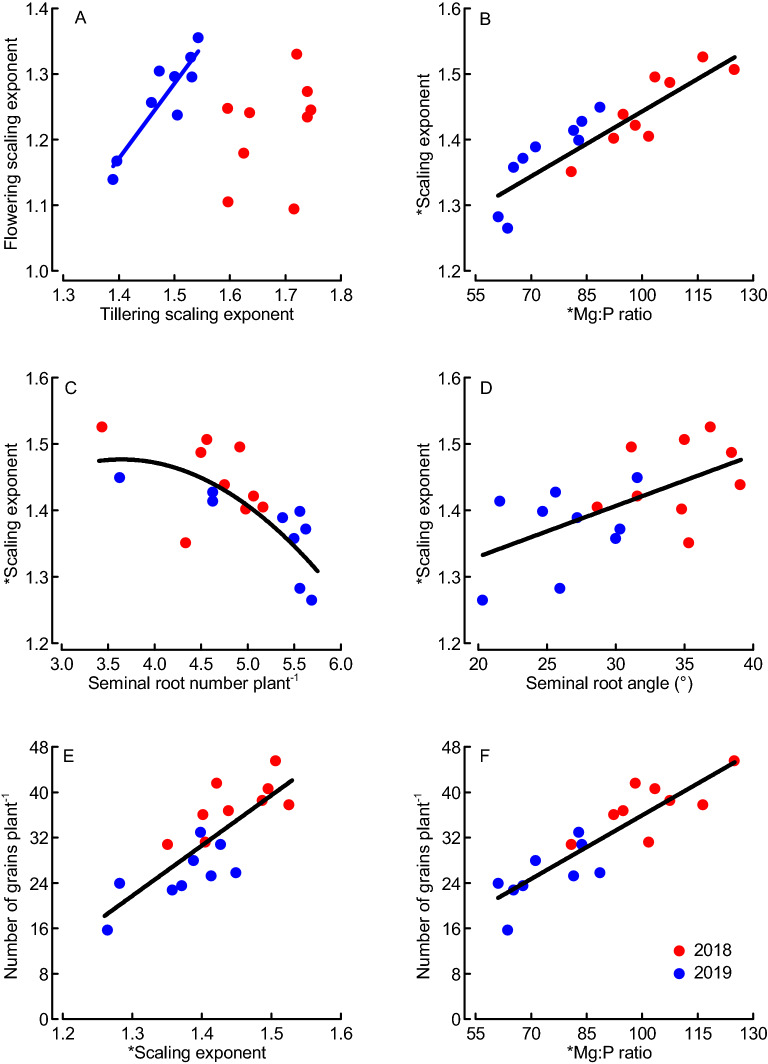
Relationships between scaling exponents (macroelements) and/or nutrient ratios at tillering (BBCH29) and flowering (BBCH65)^35^, root traits at tillering and number of grains per plant at maturity across nine wheat varieties field-grown in Sweden during two years (2018, 2019). *Indicates that scaling exponents were based on data from tillering and flowering. Regressions: y = 0.426x + 0.501, *N* = 9, *R*^2^ = 0.13, *P* = 0.345 n.s. (**A**, 2018); y = 1.148x − 0.436, *N* = 9, *R*^2^ = 0.82, *P* = 0.001 (**A**, 2019); y = 0.003x + 1.113, *N* = 18, *R*^2^ = 0.78, *P* < 0.001 (**B**); y = − 0.038x^2^ + 0.277x + 0.972, *N* = 18, *R*^2^ = 0.47, *P* = 0.002 (**C**); y = 0.008x + 1.178, *N* = 18, *R*^2^ = 0.36, *P* = 0.009 (**D**); y = 88.66x − 93.55, *N* = 18, *R*^2^ = 0.63, *P* < 0.001 (**E**); y = 0.374x − 1.48, *N* = 18, *R*^2^ = 0.77, *P* < 0.001 (**F**). Dots represent means from 4 replicates.

There was a significant relationship between RMA scaling exponents calculated for the flowering and tillering data in 2019, but not 2018 (Fig. 4A). RMA scaling exponents (macro) based on data from both tillering and flowering significantly decreased with increased seminal root number and increased with root angle (Fig. 4C,D). A 78% of the variation in scaling exponent was explained by the mean Mg:P ratio (Fig. 4B). The number of grains, which in wheat is determined already at the induction of flowering (booting stage), was strongly associated with both the scaling exponent and mean Mg:P ratio (Fig. 4E,F).

### Scaling exponents and Mg:P ratios were linked to grain yield and protein content

**Figure 5:**
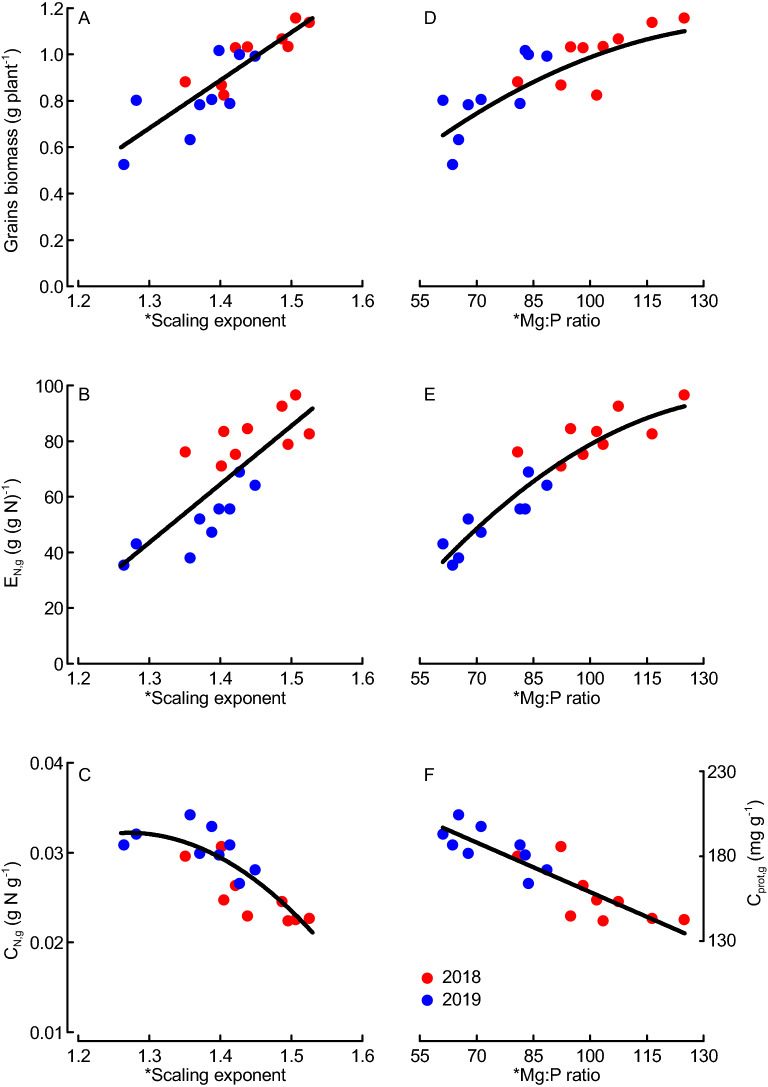
Grains biomass, grain-specific nitrogen (N) efficiency (E_N,g_) and grain N concentration (C_N,g_) or grain protein concentration (C_prot,g_) as functions of the scaling exponent for macroelements (**A** to **C**) and Mg:P ratio (**D** to **F**) for nine wheat varieties field-grown in Sweden during two years (2018, 2019). *Indicates that scaling exponents were based on data from tillering (BBCH29) and flowering (BBCH65)^35^. Regressions: y = 2.069x − 2.008, *N* = 18, *R*^2^ = 0.73, *P* < 0.001 (**A**); y = 209.9x − 229.34, *N* = 18, *R*^2^ = 0.63, *P* < 0.001 (**B**); y = − 0.166x^2^ + 0.422x − 0.236, *N* = 18, *R*^2^ = 0.68, *P* < 0.001 (**C**); y = − 6.434E − 5x^2^ + 0.019x − 0.269, *N* = 18, *R*^2^ = 0.70, *P* < 0.001 (**D**); y = − 0.008x^2^ + 2.419x − 80.15, *N* = 18, *R*^2^ = 0.88, *P* < 0.001 (**E**); y = − 0.000184x + 0.044, *N* = 18, *R*^2^ = 0.77, *P* < 0.001 (**F**); the y_2_ axes applied for (**C**) and (**F**) were adjusted according to the linear regression C_prot,g_ = 5288.5 * C_N,g_ + 23.5, *R*^2^ = 0.97, *P* < 0.001, N = 36. Dots represent means from 4 replicates.

Grains biomass was uncorrelated with mean biomass at tillering and flowering when calculated within each year; and decreasing with increasing mean plant biomass when calculated across the two years (Pearson *R* = − 0.65, *P* = 0.003, *N* = 18). Furthermore, grains biomass was inversely correlated with grain N content (C_N,g_) and protein content (C_prot,g_) (Pearson *R* = − 0.78, *P* < 0.001, *N* = 18). The grains biomass and grain-specific N efficiency (E_N,g_) increased with the RMA scaling exponent (macroelements) and Mg:P ratio; whilst the grain N and protein contents decreased with increasing RMA scaling exponent and Mg:P ratio (Fig. 5). The grains biomass assessed for the five individual plants per plot (i.e., the same plants that were used for the nutrient analyses) was a good predictor for the agronomic grain yield assessed at plot basis (see Material and Methods) when evaluated separately for each year (Supplement S2B). Increased agronomic grain yields were associated with enhanced RMA scaling exponents (macroelements) (regressions: *R*^2^ = 0.84 and *P* < 0.001 for 2018; *R*^2^ = 0.78 and *P* = 0.011 for 2019).

### Evidence for grain yield co-limitation by Mg and P

**Figure 6:**
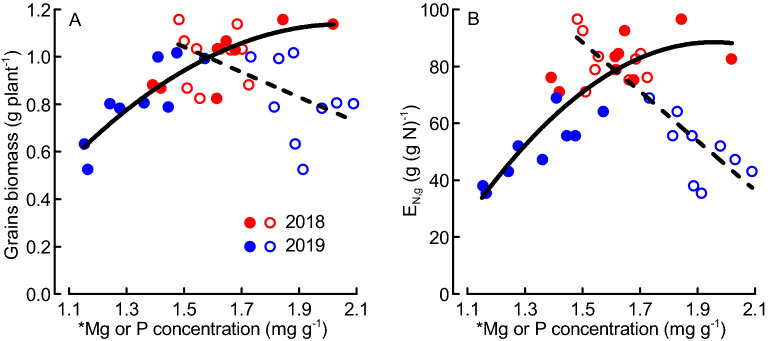
Grains biomass (**A**) and grain-specific nitrogen efficiency (E_N,g_) (**B**) as functions of the shoot magnesium (Mg, closed symbols) or phosphorus (P, open symbols) concentrations for nine wheat varieties field-grown in Sweden during two years (2018, 2019). * indicates that Mg or P concentrations were based on data from tillering (BBCH29) and flowering (BBCH65)^35^. Regressions: y = − 0.637x^2^ + 2.617x − 1.55, *N* = 18, *R*^2^ = 0.80, *P* < 0.001 (**A, solid line for Mg**); y = − 0.528x + 1.834, *N* = 18, *R*^2^ = 0.34, *P* = 0.011 (**A, broken line for P**); y = − 84.08x^2^ + 329.08x − 233.51, *N* = 18, *R*^2^ = 0.79, *P* < 0.001 (**B, solid line for Mg**); y = − 86.41x + 218.03, *N* = 18, *R*^2^ = 0.76, *P* < 0.001 (**B, broken line for P**). Dots represent means from 4 replicates.

The grains biomass and E_N,g_ steadily increased with the mean shoot Mg concentrations for tillering and flowering, but with diminishing returns above tissue Mg concentrations of ca. 1.6 mg g^−1^ (Fig. 6). In parallel, the mean shoot P concentrations significantly decreased with increasing grains biomass and increasing E_N,g_. Taken together, the shoot P concentration was much higher than the Mg concentration when low grains biomass and E_N,g_ were achieved, but fell below the Mg concentrations at grain biomass values above ca. 1 g plant^−1^ (or E_N,g_ of 80 g (g N)^−1^) and when both P and Mg concentrations were around 1.6 mg g^−1^.

## Discussion

This study is first in relating the stoichiometric volumes of different groups of nutrients (beyond N and P) to crop yields and root characteristics. In general, we found the vegetative tissue concentrations of the investigated micro- and macronutrients to increase stronger than the concentrations of N and P, which was reflected by scaling exponents > 1 for both micro- and macroelements. On average, the micronutrients accumulated much faster than N and P as reflected by scaling exponents around 3 (Fig. 2D). However, the accumulation of the micronutrients was extremely variable (reflected by high SE), because certain micronutrients such as iron (Fe) sporadically accumulated to very high values already shortly after sowing (source data at figshare). A similar pattern was reported also for early-season micronutrient accumulation in field-grown winter wheat^18^, and the great portion of variability not explained by the factors imposed here suggests that the micronutrient accumulation was controlled by other factors. Hence, the relationships between micronutrient accumulation (and scaling exponents), crop productivity and root traits cannot be explored using this data. For the macronutrients, the scaling exponents found here were higher than the scaling exponents reported for winter wheat^9^. The higher scaling exponents in spring wheat than in winter wheat indicate that the relative accumulation of macronutrients other than N and P was greater in spring wheat than winter wheat. In contrast to spring wheat, nutrient uptake in winter wheat starts already during late fall and winter without concomitant growth^18^. Moreover, periods early in the growing season with enhanced nutrient uptake rates that are not matched by proportional growth have been also reported for other crops^29^. It is therefore possible that the generally higher scaling exponents in spring wheat compared to winter wheat reflect a particularly great demand for nutrients other than N and P to reduce the risk of future co-limitation of growth. We found P to accumulate slower than N, which is in agreement with the general pattern observed for many plants^7,9,10^. The scaling exponent between N and P found in our study is low compared to the ones reported for other plants, suggesting that P possibly was limiting growth and yield in our study. However, the scaling exponents between N and P reported for other plants are usually interpreted in relation to vegetative plant growth but not crop (grain) yields; and vegetative biomass was uncorrelated with grains biomass in our study and other investigations^30,31^. In addition, higher grains biomass was associated with lower P concentrations in the growing crop (Fig. 6), suggesting either that P was not strongly limiting grain yields in our study, or that low P concentrations were partly compensated for by other nutrient(s) in a synergistic manner as was reported for some nutrients^32^.

### Scaling exponent and Mg:P ratio as predictors for grains number and yield in wheat

**Figure 7:**
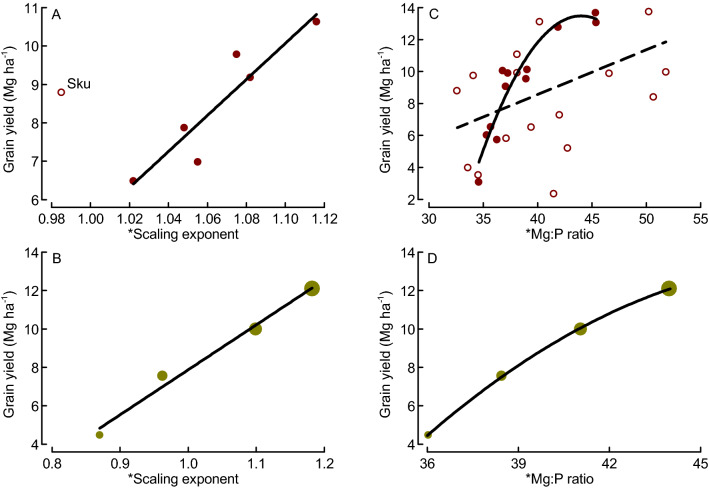
Mean winter wheat grain yields as function of scaling exponent for macroelements (**A**, **B**) and Mg:P ratio (**C**, **D**) plotted for various locations in Southern and Central Sweden (**A**, **C**) or across different fertilization levels at the same locations (**B**, **D**; increasing symbol size indicates increasing fertilizer level i.e. 0, 80, 160 and 240 kg N ha^−1^) based on the original data by Hamnér et al. (2017)^19^**.** * indicates that scaling exponents were based on data from stem elongation (BBCH37) and flowering (BBCH65)^35^. Regressions: y = 46.92x − 41.55, *N* = 6, *R*^2^ = 0.86, *P* = 0.007; the data point for the location “Skultuna” (**Sku**) was not considered in the regression (**A**); y = 23.404x − 15.530, *N* = 4, *R*^2^ = 0.98, *P* < 0.001 (**B**); y_1_ = 0.28x − 2.64, *N* = 28, *R*^2^ = 0.21, *P* = 0.015 (all data, **C,** **broken line**) and y_2_ = − 0.103 × 2 + 0.777x − 20.87, *N* = 12, *R*^2^ = 0.90, *P* < 0.001 (only data from the locations Grillby, Nybble and Strömsholm, **C, solid line**) (**C**); y = − 0.052x^2^ + 5.126x − 112.51, *N* = 4, *R*^2^ = 1.00, *P* = 0.016 (**D**). Dots represent means of 4 fertilization treatments and 4 replicates (A), 7 locations and 4 replicates (B, D) or 4 replicates (C).

We found the scaling exponent for macro nutrients and the Mg:P ratio in the growing crop, but not the vegetative biomass itself, to be strong determinants of grains biomass and yield (Fig. 5). All previous studies interpreted element scaling exponents in relation to the concurrent biomass, then implying that the scaling relations are both cause and consequence of element and biomass accumulation processes occurring simultaneously^7^. We have here related the element scaling exponents and ratios assessed during the main period of vegetative biomass growth to the grains biomass and yield formed after the main period of vegetative growth, which enables us to interpret them as causes for (or predictors of) grain yield. Historically, yield gains in wheat have resulted from increases in harvest index and not vegetative biomass accumulation^30^, and vegetative biomass is therefore expected to be a poor predictor for grains biomass in wheat as was also found in our study. Also the early growth of leaves and shoots (early vigor), which is considered important for nutrient uptake and yield formation of spring-sown crops especially under the short growing seasons at higher latitudes^17^, was not a reliable predictor of grains biomass in our study (Fig. 3B). In contrast to vegetative biomasses, the scaling exponent for macronutrients, which here was explained by the Mg:P ratio in the growing crop, were found to be good predictors for grains biomass when assessed both within and across years. We focused our analysis on the grains biomass per plant, because it was important for consistency to use the same plant material for the assessments of biomasses and nutrient contents. Grains biomasses assessed at individual-plant level often do not well reflect the plot-based agronomic grain yields (Mg ha^−1^)^33^. In our study, the predictive power of grains biomass, and notably also scaling exponent, for forecasting agronomic grain yield was high when evaluated separately for the two years (*R*^2^ values between 0.69 and 0.85; Supplement S2B). Hence, the pattern seen at individual-plant level reflected the overall pattern of agronomic grain yields when assessed within years. Enhanced grains biomass (or agronomic yield) has often been reported to result mainly from enhanced number of kernels^30^, which in turn is determined already at the induction of flowering (booting stage). In our study, the scaling exponent for macronutrients and particularly the Mg:P ratio in the growing crop were good predictors of the kernel numbers (Fig. 4E,F), which provides a mechanistic explanation linking the vegetative tissue nutrient concentrations to grains biomass. Both Mg and P are heavily involved in various photosynthetic processes, and low Mg concentration has been reported to reduce N conversion efficiency (i.e., the grain production per plant-internal N)^16,34^. Our observation of strongly increasing grain-specific N efficiency (E_N,g_) with Mg:P ratio (Fig. 5E) could therefore indicate a synergistic mechanism of Mg and P in supporting photosynthetic N use and ultimately biomass and grain production. The strong inverse relationship between grain protein (or N) contents and the scaling exponent (particularly the Mg:P ratio) observed here reflects the negative relationship between wheat grain yield and protein content observed also by others^30^. When we plotted the grains biomass (and E_N,g_) separately against the concentrations of Mg and P, evidence emerged for Mg being limiting E_N,g_ at lower grains biomass, whilst P being increasingly limiting at grains biomass values above ca. 1 g plant (or concentrations above 1.6 mg g^−1^; Fig. 6). The Mg concentrations observed here are within the critical range of 1 to 2 mg (g biomass)^−1^ reported for leaves of a large group of plants including wheat^16^, but our results highlight the possible role of functional links between different elements (here Mg and P) in co-limiting yield formation which cannot be described by threshold concentrations for single elements^3–5^. The suggested mechanism of a Mg–P synergism and co-limitation of grains biomass was derived from a field experiment, and we expect a strong influence of the soil Mg and P availability on the results. In spite of P fertilization, the soil of our study site was characterized by low P availability, while Mg availability was high (Table 1). This is probably important for the interpretation of our results: The Mg:P ratio appears to be a good predictor of grains biomass mainly under high-Mg and low-P conditions. Re-visiting already published data from winter wheat grown in seven field sites and under various fertilizer conditions^19^ provided evidence for both generalizability and limitations of our results. The two datasets combine grain yield data ranging from the relatively low values in this study (1.4 to 3.8 Mg ha^−1^) to the considerably higher values that were achieved with winter wheat (< 0.5 to 13.7 Mg ha^−1^)^19^. First, these data showed significantly increasing grain yields with increasing scaling exponent for macronutrients (Ca, K, Mg, S) across most sites and for all fertilizer levels (Fig. 7A,B). The only exception was one location (Skultuna) with high soil P availability resulting in high plant P concentrations and ultimately low scaling exponents along with rather high grain yields. Second, grain yields increased significantly with the Mg:P ratio in the growing crop across all sites and fertilizer levels, and the predictive power increased considerably when the regression was calculated only for those three locations in which the extractable Mg content in soil was high, i.e. above 150 g kg^−1^ according to the original publication^19^ (Fig. 7C,D). Taken together, our results from spring wheat and the re-visited data from winter wheat demonstrate that Mg–P synergism and co-limitation might be a general pattern relevant to wheat growth in many locations, but the pattern depends on the availability of the elements critical for crop yield formation. In soil conditions with nutrient availabilities different from the ones considered here, co-limitation of growth by elements other than Mg and P might occur. The calculation of RMA scaling exponents as they were applied here, followed by an evaluation of specific element ratios can be a useful procedure in the exploration of nutrient co-limitation in field-grown crops.

### Root traits influencing the accumulation of P and Mg

Apart from soil availability, plant uptake characteristics affect the acquisition of nutrients by plants. Magnesium and P were here identified as important in affecting harvestable wheat yields, and it is therefore of great interest to link Mg and P contents to those plant traits that determine the acquisition of these elements. Phosphorus is less mobile and typically has a shallow distribution in soil^26^, and we found an increased P:N ratio with seminal root number (at tillering) beyond ca. 4 roots plant^−1^ (Fig. 3E). The inverse relationship between the seminal root number and root angle suggests a functional link between topsoil rooting and the uptake of less mobile nutrients such as P^20,22,27^. Steeper root angles, here and previously^25^ used as an indicator for deeper roots, were associated with higher scaling exponents and Mg:N ratios in our study. It is therefore possible that deeper roots facilitate the acquisition of Mg and other nutrients with a typically more even vertical distribution across the soil profile^26^. Root traits are generally difficult to quantify especially under field conditions, and links between the uptake of specific nutrients and root traits challenging to establish. Therefore, our results are novel as they for the first time show those links in field-grown wheat.

### Opportunities for crop management and breeding

Increasing scaling exponents with increasing grain yields imply a more than proportionally enhanced demand for nutrients other than N and P to achieve high grain yields. The Mg–P synergism proposed here adds further evidence to the previously made suggestion of Mg fertilization as a means to enhance wheat yields^15^, and specifies the conditions in which Mg fertilization should be considered, i.e., in fields with low soil Mg supply in relation to the P supply. Critical concentrations of single nutrients as indicators for optimized crop fertilization should be interpreted with care, because they do not consider the possible synergistic (or antagonistic) effects between nutrients^32^, here shown for Mg and P. Wheat breeding research has attempted to increase the efficiency of P uptake, in particular early in the growing season^12–14^. Our results indicate that targeting only increased P acquisition is not sufficient, because increased P acquisition capacity probably needs to be accompanied by greater capacity to accumulate Mg. In addition, our results highlight candidate root traits (i.e., number and angle of seminal roots) for increased P and Mg uptake in wheat breeding, as a significant proportion of the observed trait variation was caused by variation between varieties (e.g. Supplements S1, S3). Analysis of phenotypic and genetic correlation of root traits and other plant traits related to nutrient uptake and grain yield will form the basis for breeding towards nutrient efficient wheat.

## Methods

### Study site

The study was carried out during the 2018 and 2019 growing seasons in a field experiment near Uppsala, Central Sweden (59° 45′ N, 17° 42′ E). The soil at the experimental site is a Cambisol formed on postglacial sediments. The soil texture is a silty clay, and information about soil properties, nutrient status and fertilization treatment is summarized in Table 1. Climate in Uppsala is boreal-temperate and the growing season normally lasts from April to October. Summer (May and July) 2018 was drier than normal with mean temperatures higher than the long-term mean, whereas summer 2019 was wetter and cooler than 2018 (Fig. 1). Due to an extended dry period (without any precipitation) that occurred in 2018, artificial irrigation of ca. 10 mm of water was applied 28 and 34 days after sowing, approx. at the beginning of stem elongation (BBCH 30).

### Experimental design

The field experiment was established in spring 2018 with a randomized split-plot design (main-plot factor soil treatment, i.e., compaction vs. non-compaction; and split-plot factor wheat variety) with four replicates and individual plot dimensions of 12 m × 2 m. Soil compaction was carried out in April 2018 by double track-by-track passing using a front loader with four wheels and an average wheel load of 42 kN. To ensure crop establishment, the surface of the compacted soil was loosened to a depth of approximately 50 mm with a surface cultivator before sowing. In 2019, the same soil compaction areas were used as in the previous year and re-compacted using the same method. The position of the variety plots was re-randomized. The following nine spring wheat varieties were grown during both years: ‘KWS Alderon’ (‘Alderon’, Germany, KWS W185), ‘Bjarne’ (Sweden, NK 97520), ‘Boett’ (Sweden, SW 71034), ‘Dacke’ (Sweden, W 26267), ‘Diskett’ (Sweden, SW 45456), ‘Happy’ (Sweden, SW 91003), ‘Quarna’ (Switzerland, CH 21112283), ‘Rohan’ (Sweden, SW 01198), and a landrace originating in Dalecarlia, Sweden (‘Dala landrace’). Conventional tillage system including ploughing and tine harrowing was performed. Wheat seeds were sown on 10th May 2018 and 23rd April 2019. Seed rates were 550 seeds m^−2^, as common in the region. Final harvest of the central plot area (6 m × 2 m) was done on 17th August 2018 and 23rd August 2019, respectively, with a combine harvester to assess agronomic grain yield (Mg ha^−1^, Supplement S2).

### Plant sampling across developmental stages

Five individual plants were selected within homogeneous areas of each entire plot at the crop developmental stages tillering (BBCH29)^35^, flowering (BBCH65) and spike maturity (BBCH89) in 2018 and 2019. The selected plants were cut with scissors at approximately 15 mm above ground, oven-dried at 65 °C for 48 h and their biomasses determined. Only at crop maturity (BBCH89), the spikes of the sampled plants were manually threshed (Smooth Chopper 6948A-2, Tupperware, USA) and grains biomasses determined after oven drying. Roots were sampled only at crop tillering (BBCH29). Four plants were selected from homogeneous areas of each plot. Intact root crowns were excavated down to a depth of approximately 0.2 m using a shovel. All excavated root crowns were washed and the numbers of seminal roots counted as described by others^36,37^. The angles between the outermost seminal roots and the soil surface were measured along an arc with a 50 mm radius^37^. Only the samples of the above-ground plant parts were further processed for nutrient analysis. These samples (straw and grains separated at maturity) were ground in a stainless steel grinder to pass a 1-mm mesh before nutrient element analysis. The N concentrations were analyzed on a LECO CNS/2000 analyzer using a standard method (SS-ISO13878). The contents of P, K, Ca, Mg, S, Mn, Fe, Zn and Cu were extracted using 32.5% Nitric Acid on a heat block and concentrations were determined using ICP-AES technique (Spectro Blue FMS 26, Spectro Analytical Instruments, Kleve, Germany) by applying internal standardization protocols (protocol number SS028311).

### Calculations of RMA scaling exponents and grain-specific N efficiency

Scaling exponents (α) for N vs. P concentrations were calculated as [P] = β[N]^α^. For the other elements, two stoichiometric volumes (V) based on realized niches were used^9^; the first was defined by the product of the plant N and P concentrations (VNP), and the second was defined by the product of other nutrient concentrations (VOth). VOth was calculated separately for macroelements (VOth_macro_; Ca, K, Mg, S) and microelements (VOth_micro_; Cu, Fe, Mn, Zn). Calculations of scaling exponents were based either on data from single sampling occasions, i.e. tillering or flowering; or including all data from both sampling occasions (then marked with an asterisk in the figures). All regressions for the stoichiometric volumes were calculated as reduced major axes (RMA; SPSS version 26 codes on figshare) using ln-transformed values as is conventional in this type of studies^10^. The grain-specific N efficiency (E_N,g_; g (g N)^−1^)) was calculated separately for individual replicates as the ratio between the grains biomasses and the weighted mean total N contents in the above-ground plant biomass at crop tillering, flowering and maturity^5,28^.

### Statistical analysis

The experimental unit was a replicate plot, and the means from the five plants sampled within each plot were used for all statistical analysis. The SPSS version 26 procedure Linear Mixed Model was used for calculating probabilities of significant differences in various plant traits and element concentrations for fixed effects of year (2018, 2019), treatment (compacted and non-compacted soil), variety, block, and the interactions between year, treatment and variety; and random effect of mainplot (Supplement S1, codes on figshare). Correlation and regression analyses were performed based on the variety means from all replicate plots and using the standard procedures in SPSS version 26. The SPSS version 26 procedure CATPCA was used to group the samples according to Principal Components Analysis (PCA) and relate the grouping to the supplementary variable year (representing weather). Samples were attributes (mean trait values from four replicates) measured on nine wheat varieties grown during two years; the samples were defined as numeric (continuous) variables, and the supplementary variable was defined categorical (Supplement S3, codes on figshare).

The study complies with relevant institutional, national, and international guidelines and legislation.

## Supplementary Information

Supplementary Information.

## Data Availability

All data used in this study are available at figshare (10.6084/m9.figshare.c.5172155). The nutrient concentration and grains biomass source data underlying all figures and tables are also available on the above web page.
