# Supplementary material for: Evidence for magnesium–phosphorus synergism and co-limitation of grain yield in wheat agriculture
Source: Sci Rep. 2021 Apr 27;11:9012. doi: 10.1038/s41598-021-88588-8 (PMC8079383; doi:10.1038/s41598-021-88588-8)

Supplement to:

Evidence for magnesium-phosphorus synergism and co-limitation of grain yield in wheat agriculture

Martin Weih, Hui Liu, Tino Colombi, Thomas Keller, Ortrud Jäck, Pernilla Vallenback, Anna Westerbergh

Supplement S1. Linear Mixed Model analysis results for fixed effects of year (2018, 2019, *df* = 1), treatment (compacted and non-compacted soil, *df* = 1), variety (*df* = 8), block (*df* = 3), and the interactions between year, treatment and variety; and random effect of mainplot (*df* = 7) on various traits assessed for nine wheat varieties field-grown in Sweden. ***F*** critical value for F-statistics, ***P*** significance (significant: $P < .050$ underlined), ***df*** degrees of freedom. Mainplot was included as a random effect to account for the varieties having been assigned to different plot positions in the two years

Trait	Year (Y)		Treatment (T)		Variety (V)		Block		Y x T		Y x V		T x V		Y x T x V	
	F	*P*	*F*	*P*	*F*	*P*	*F*	*P*	*F*	*P*	*F*	*P*	*F*	*P*	*F*	*P*
G_B	25.31	.000	13.90	.034	13.77	.000	.87	.543	5.35	.023	.63	.752	.98	.453	1.13	.353
G_Y	102.19	.000	3.81	.146	38.09	.000	11.04	.040	9.56	.003	4.68	.000	.96	.474	.76	.642
G_{no}	118.23	.000	11.05	.044	11.34	.000	.80	.571	6.90	.010	.91	.515	1.06	.396	1.21	.298
R_{no}	43.67	.000	3.60	.154	21.36	.000	.65	.636	.62	.434	.703	.689	.48	.867	1.12	.359
R_a	62.94	.000	6.23	.088	3.95	.000	.24	.865	.00	.991	1.00	.444	.74	.657	.58	.792
$E_{N,g}$	98.10	.000	1.76	.276	5.58	.000	1.83	.316	20.81	.000	1.36	.222	.41	.913	1.88	.072
$C_{N,g}$	873.8	.000	6.20	.089	75.31	.000	.69	.616	.73	.395	9.17	.000	.80	.604	.99	.448
NC_{till}	250.31	.000	27.90	.013	8.36	.000	5.27	.103	.26	.610	1.33	.237	.31	.963	.43	.900
CaC_{till}	19.13	.000	.80	.438	2.61	.012	3.02	.194	.15	.698	.64	.747	1.05	.408	.84	.567
KC_{till}	367.06	.000	26.27	.014	4.81	.000	.69	.617	.00	.962	2.27	.028	1.34	.232	1.13	.350
MgC_{till}	67.16	.000	.33	.605	2.74	.009	3.12	.187	.01	.908	.62	.756	.83	.579	.90	.521
PC_{till}	130.47	.000	5.12	.109	2.26	.029	5.82	.091	10.32	.002	.76	.639	.14	.997	.50	.854
SC_{till}	2.88	.093	3.17	.173	4.82	.000	1.85	.313	.75	.387	1.03	.417	.36	.937	.96	.469
NC_{flow}	11.59	.001	1.96	.256	4.25	.000	1.19	.444	5.83	.018	1.34	.231	.41	.914	.61	.769
CaC_{flow}	22.74	.000	6.50	.084	9.00	.000	16.00	.024	2.09	.151	1.21	.301	1.07	.390	1.00	.441
KC_{flow}	86.40	.000	1.16	.360	8.54	.000	2.84	.207	2.32	.131	1.56	.147	.50	.852	2.09	.043
MgC_{flow}	.01	.921	4.71	.118	15.94	.000	6.70	.076	6.16	.015	1.69	.110	.78	.621	1.90	.068
PC_{flow}	13.64	.000	16.01	.028	7.23	.000	1.65	.345	17.50	.000	1.13	.348	.258	.978	.849	.562
SC_{flow}	2.88	.093	3.17	.173	4.82	.000	1.85	.313	.75	.387	1.03	.417	.36	.937	.96	.469

$\mathbf{G_B}$ Grains biomass (assessed on 5 indiv. plants), $\mathbf{G_Y}$ Agronomic grain yield (assessed on plot basis), $\mathbf{G_{no}}$ Number of grains per plant, $\mathbf{R_{no}}$ Seminal root number, $\mathbf{R_a}$ Seminal root angle, $\mathbf{E_{N,g}}$ Grain-specific N efficiency, $\mathbf{C_{N,g}}$ Grain N concentration, **NC** N concentration, **CaC** Calcium concentration, **KC** potassium (K) concentration, **MgC** Magnesium concentration, **PC** Phosphorus concentration, **SC** Sulfur concentration; subscripts ***till*** tillering (BBCH29) and ***flow*** flowering (BBCH65) stage

Supplement S2. Mean (± 1 SD, n = 8) agronomic grain yields (Mg ha^{-1}) (A) and relationships between the plot-based values of agronomic grain yield and individual-based values of grains biomass (g plant^{-1}) (B) for nine spring wheat varieties field-grown in Sweden during two years (2018, 2019). Linear regressions in B: y=2.738x-0.478, N = 9, R^2 = 0.85, P < 0.001 (2018); y=3.221x-0.005, N = 9, R^2 = 0.69, P = 0.006 (2019). The agronomic yields of the high-yielding varieties reported here (e.g., 'Alderon', 'Happy') were of a similar magnitude compared to the commercial grain yields for spring wheat achieved in Sweden (means 2.8 and 4.5 Mg ha^{-1} in 2018 and 2019, respectively; available at www.scb.se).

A

Variety	*2018*	*2019*
'Alderon'	2.92 ± 0.36	3.80 ± 0.40
'Bjarne'	1.75 ± 0.25	2.31 ± 0.36
'Boett'	2.05 ± 0.25	2.40 ± 0.50
'Dacke'	2.04 ± 0.23	2.36 ± 0.43
'Dala landrace'	1.51 ± 0.21	1.86 ± 0.18
'Diskett'	2.21 ± 0.21	2.27 ± 0.36
'Happy'	2.21 ± 0.21	2.85 ± 0.56
'Quarna'	1.42 ± 0.22	2.55 ± 0.44
'Rohan'	2.23 ± 0.22	2.54 ± 0.54

B

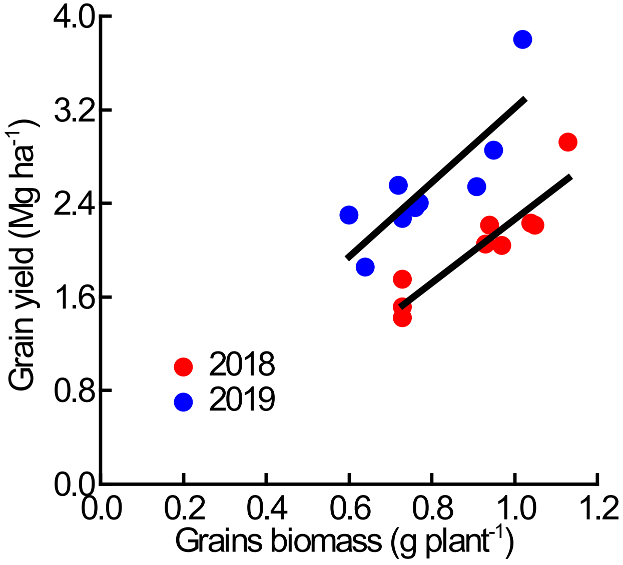

Supplement S3. Grouping of samples (A) and corresponding variable component loadings (B) according to Principal Components Analysis (PCA). Samples are attributes (mean trait values) measured on nine wheat varieties field-grown in Sweden during two years (2018 red colors, 2019 blue colors), i.e. the variables shown in (B). Eigenvalues 7.47 for dimension 1 (explanatory power 75 %), and 1.41 for dimension 2 (explanatory power 14 %). Varieties **Ald** 'Alderon', **Bja** 'Bjarne', **Boe** 'Boett', **Dac** 'Dacke', **Dal** 'Dala landrace', **Dis** 'Diskett', **Hap** 'Happy', **Qua** 'Quarna', **Roh** 'Rohan'. Variables: $\mathbf{B_T}$ Plant biomass plant^{-1} at tillering, $\mathbf{B_G}$ Grains biomass plant^{-1}, $\mathbf{R_{no}}$ Seminal root number plant^{-1}, $\mathbf{R_a}$ Seminal root angle, $\mathbf{E_{N,g}}$ Grain-specific nitrogen (N) efficiency, $\mathbf{C_{N,g}}$ Grain N concentration, $\mathbf{P{:}N_{till}}$ P:N ratio at tillering, $\mathbf{Mg{:}N_{till}}$ Mg:N ratio at tillering, ***Mg:P** Mean (tillering and flowering) Mg:P ratio, ***ScExp** scaling exponent (macroelements) based on data from tillering and flowering; **Year** (supplementary variable).

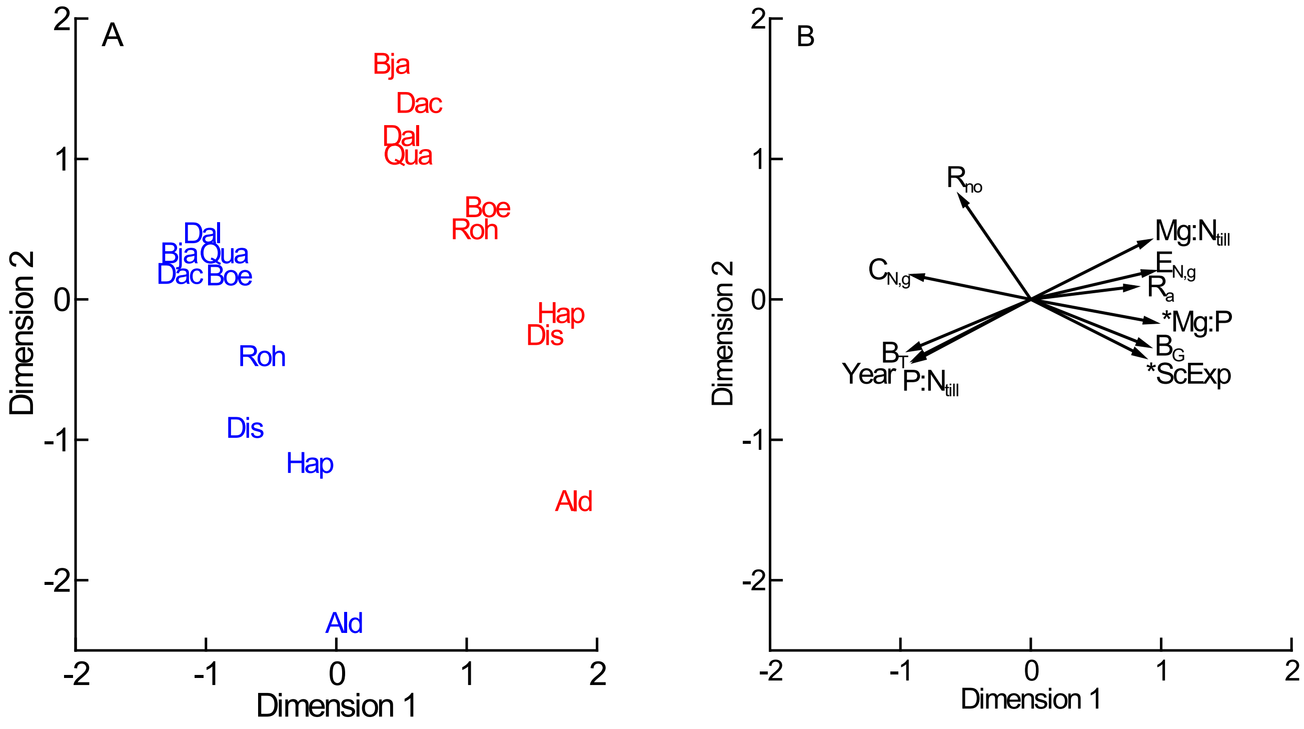

Supplement: Supplementary file 1 — Supplementary Information. [file 41598_2021_88588_MOESM1_ESM.pdf]
